# Pearl shape classification using deep convolutional neural networks from Tahitian pearl rotation in *Pinctada margaritifera*

**DOI:** 10.1038/s41598-023-40325-z

**Published:** 2023-08-12

**Authors:** Paul-Emmanuel Edeline, Mickaël Leclercq, Jérémy Le Luyer, Sébastien Chabrier, Arnaud Droit

**Affiliations:** 1 Département de médecine moléculaire, Faculté de Médecine, Université Laval, Québec, Canada; 2 Géopole du Pacifique Sud, Université de Polynésie Française, Faa’a, Tahiti French Polynesia; 3 Institut Français de Recherche pour l’Exploitation de la Mer, Vairao, Tahiti French Polynesia

**Keywords:** Biomineralization, Data acquisition, Image processing, Machine learning

## Abstract

Tahitian pearls, artificially cultivated from the black-lipped pearl oyster *Pinctada margaritifera*, are renowned for their unique color and large size, making the pearl industry vital for the French Polynesian economy. Understanding the mechanisms of pearl formation is essential for enabling quality and sustainable production. In this paper, we explore the process of pearl formation by studying pearl rotation. Here we show, using a deep convolutional neural network, a direct link between the rotation of the pearl during its formation in the oyster and its final shape. We propose a new method for non-invasive pearl monitoring and a model for predicting the final shape of the pearl from rotation data with 81.9% accuracy. These novel resources provide a fresh perspective to study and enhance our comprehension of the overall mechanism of pearl formation, with potential long-term applications for improving pearl production and quality control in the industry.

## Introduction

The pearl industry is a vital sector in French Polynesia, representing a major economic pillar for the region. In 2021, the production of Tahitian pearls was estimated at around 10 million pearls per year, contributing to nearly 50% of French Polynesia’s exports^1^. This dynamic industry employs over 3,000 people, primarily in the atolls of the Tuamotu and Gambier archipelagos and generates an estimated annual revenue of 4.75 billion XPF. Pearl farming is also crucial for the sustainable development of remote islands, promoting local economic growth while preserving the environment and marine resources. Understanding the process of Tahitian pearl formation is thus critical for achieving quality and sustainable pearl production in French Polynesia. By gaining insights into the biological, environmental, and cultural factors that influence pearl development, researchers can identify best practices for optimizing pearl quality while minimizing the ecological impact of pearl farming. Consequently, this knowledge can inform policies and management strategies aimed at promoting the long-term viability of the Tahitian pearl industry in French Polynesia.

Oysters are bivalve mollusks widely distributed in marine and estuarine environments. They are commonly found in shallow coastal waters and are often farmed for their edible meat and their ability to produce pearls. One species of oyster that is particularly well-known for its ability to produce pearls is the black-lipped pearl oyster, *Pinctada margaritifera* (Linnaeus, 1758). This species is common in the coral reefs of the Indo-Pacific area^2^, and is the main source of Tahitian pearls, also known as black pearls.

Pearls are the only gemstones produced by living creatures^3^. Natural pearls are rare, so to stimulate nacre production, also known as mother-of-pearl^4^, a foreign body can be intentionally introduced into an oyster^5^. Cultured pearls are created through a grafting process in which a small piece of mantle tissue from a donor oyster (the *saibo*), along with a nacre bead known as the nucleus, is inserted into the gonad of the recipient oyster. Upon insertion, the outer epithelial cells of the graft multiply and form a pearl sac around the nucleus. The pearl sac then begins to deposit layers of nacre onto the nucleus, marking the start of the pearl’s formation. It takes 12 to 18 months of cultivation for the pearl to develop a thick enough layer of nacre to be sold^6^. The formation of the pearl is achieved by the superposition of nacre layers around the nucleus at a rate of 3 to 4 per day^7,8^. The secreted nacre is primarily composed of calcium carbonate ($CaCO_3$) crystals, known as aragonite, that are arranged in a brick-and-mortar structure, also called aragonite tablets. Tahitian pearls can exhibit a wide range of phenotypes, including variations in size, shape, color, and luster^9^.

In recent years, several studies have focused on understanding the factors and genes that contribute to the quality and characteristics of Tahitian pearls, highlighting the environmental and genetic factors that can influence pearl quality^10,11^, as well as the Mendelian inheritance of rare flesh and shell colors in *P. margaritifera* and how it controls the color of the pearls^12^. Among these studies, it has been found that the growth fronts of nacre on Tahitian pearls can be observed at the microscopic level and may take the form of spirals or targets^9^. The shape of these lines, similar to fingerprints, suggested that the pearl moves within the pearl sac. Cartwright et al.^9^ then proposed a theory of pearl rotation based on the idea that forces during the deposit of aragonite tablets can cause pearl movement. It is believed that the orientation of aragonite layers on the surface gives momentum to the pearl during its growth, leading to movement, and that different rotational movements may occur, depending on the presence or absence of defects. Further verification of this theory and analysis of pearl rotation was still necessary to determine its potential effects on the final phenotype of the pearl.

In 2015, evidence of pearl rotation in the pearl sac of *P. margaritifera* was obtained using a magnet inserted in the nucleus of a grafted pearl oyster, and magnetic field sensors^13^. A hypothetical link between the rotation and the final shape of the pearl had been suggested, and the effects of temperature on rotation have also been studied with the same device^14^, but the device used was not precise enough to allow reliable conclusions.

Acknowledging the necessity for more accurate and reliable methods to investigate pearl rotation and its relationship with the final shape of the pearl, we took advantage of the field of deep learning, specifically deep neural networks (DNNs). These networks have become the standard approach for various classification tasks, largely due to their exceptional performance in image recognition challenges. This success can be attributed to the availability of extensive, well-annotated datasets like the ImageNet dataset^15^, as well as the use of transfer learning. Transfer learning enables the utilization of pre-trained neural network models to enhance performance on related tasks. In our study, we apply this technique to our data, evaluating the link between pearl rotation and its final shape.

This paper presents an innovative, accurate and reliable device to study pearl rotation, as well as initial experiments and findings. Our study aims to better understand the rotation of Tahitian pearls during their formation and its relationship with the attributes of the pearl, especially its shape. We present the first rotation follow-up from graft to harvest, with continuous acquisitions for 1 year on multiple pearls (n = 52 oysters).

Through transfer learning and deep convolutional neural networks, using the VGG-16 architecture^16^, we establish a strong correlation between the rotation patterns of the pearl during its formation and its final shape. For all our pearls, we demonstrate an average rotation speed of 0.69 ± 0.13° min^−1^, and we highlight that in every individual case, the absence of rotation during formation was associated with no aragonite deposition around the nucleus. This confirms the significant role of rotation in detecting aragonite deposits and, consequently, in monitoring the formation of a Tahitian pearl. We thus provide a first rotation database for the pearl, as well as a model to predict the final shape of the pearl from new rotation data. This non-invasive method of rotational tracking allows for the monitoring of pearl grafting and development without sacrificing the pearl oysters. It has potential applications in a variety of studies, including those focused on understanding the factors that influence pearl quality, optimizing pearl production in the pearl industry, and studying the mechanisms of pearl formation. By tracking pearl rotation and other characteristics during development, we may be able to gain new insights into the complex process of pearl formation and identify new ways to improve pearl quality.

## Material and methods

In this section, we present our complete methodology for studying pearl rotation. The first part of our approach involves the creation of magnetized nuclei, which are essential for our experiments. In the second part, we introduce our data acquisition device that allows us to collect high-quality data. To ensure accurate results, we describe our calibration process in the third part. The fourth part details the different grafts that have been made for our experiments. In the fifth part, we describe the process of data acquisition and processing, with dedicated software that we have developed. The classification model used to predict the final shape of the pearl from its rotation data over time is detailed in the sixth part. Overall, our methodology provides a comprehensive approach to studying pearl rotation and offers valuable insights into their behavior and demonstrates a direct link between the rotation patterns and the final shape of the pearl.

### Preparation of the magnetized nucleus

To perform all our experiments, magnetized nuclei were made manually in the following steps:

1. Spherical nacre beads (2.1 bu, Imai Seikaku Co. Ltd, Sumoto, Japan, made from the shells of the freshwater mussel *Amblema* sp.) and cylindrical neodymium magnets (5-mm diameter, 1-mm thick, N52 magnetic strength, Supermagnete, Gottmadingen, Germany) were commercially purchased.
2. The beads were drilled for 5.7mm in the parallel direction to the rings observed on the surface, and the magnets were inserted at the bottom, so that the magnet was inserted exactly in the middle of the nuclei.
3. The holes were covered with dental resin (SmileFind, Tooth Gem Kit) and the nuclei were exposed to UV light for 1 h (254 nm, 10J).
4. Sander was used to smooth the dental paste and restore a spherical shape to the nuclei.

**Figure 1:**
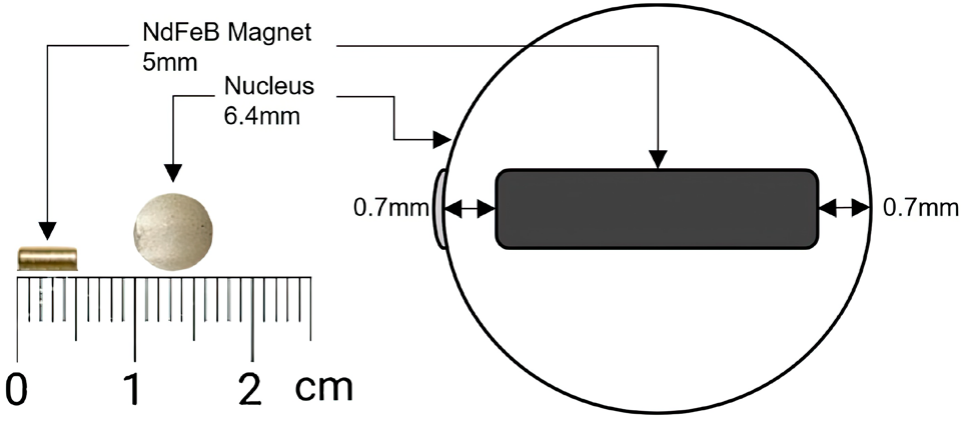
Magnet and nucleus used, to scale, and schematic drawing of the nucleus and inserted magnet.

The result is shown Fig. 1. It is crucial to restore the final spherical shape of the nucleus to avoid any impact on the graft. To obtain larger pearls, larger nuclei could have been used, but with a greater risk of rejection on the graft^17^.

### Magnetometer system

**Figure 2:**
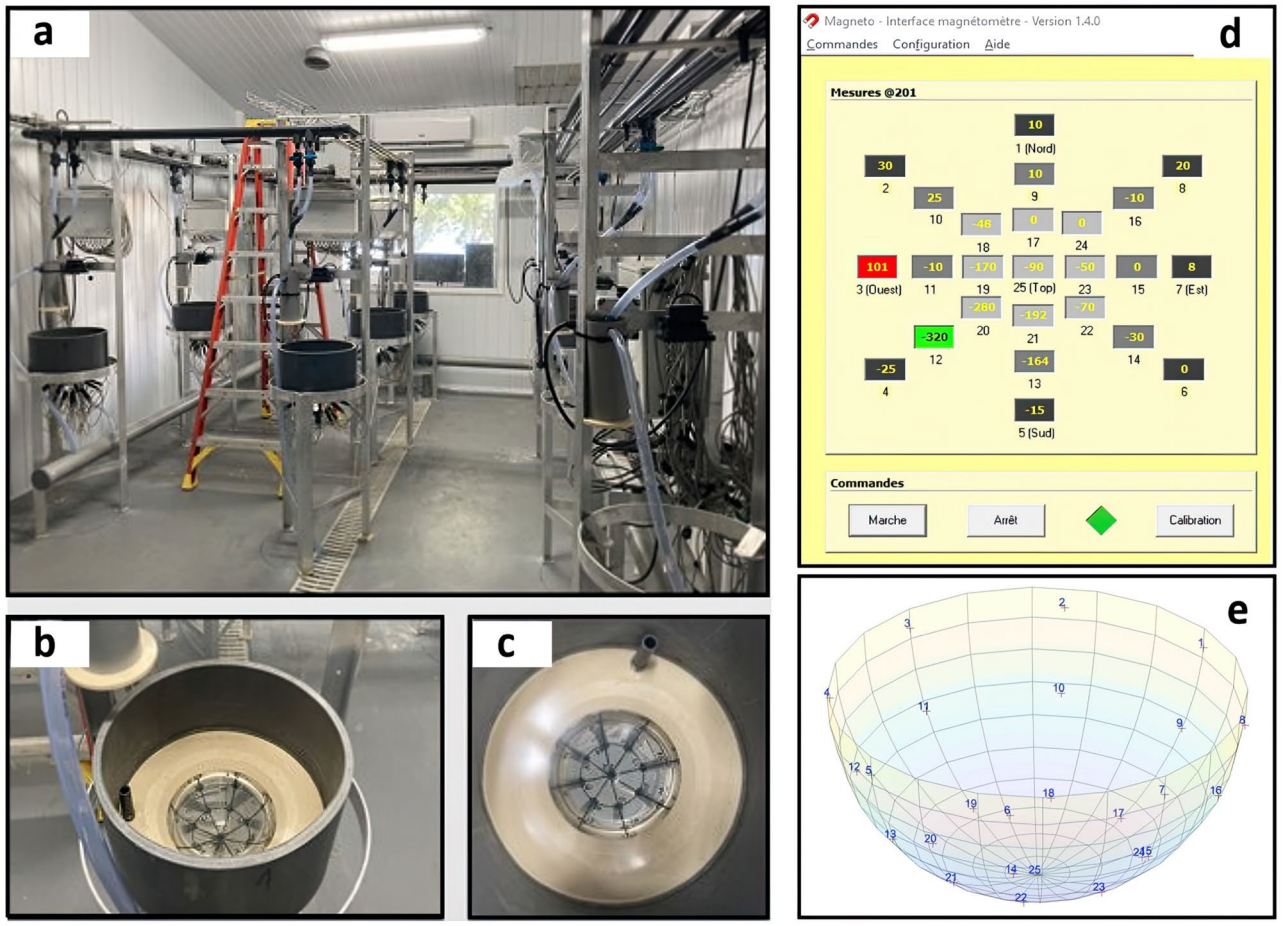
Description of the magnetometer system. (**a**) Full dedicated room with 8 domes. (**b,c**) View from the top of a dome and its 25 associated magnetic sensors. (**d**) Direct magnetic field data acquisition interface, developed by Vega Industrie. (**e**) Theoretical representation of a dome via Matlab^18^ with each of the associated sensors.

Based on the preliminary work and experimental setup of Gueguen et al, in 2015, which proved the rotation of the pearl^13^, a dedicated room has been set up at Ifremer, Vairao, Tahiti (Fig. 2a–c). The room is composed of eight domes (4.2 L), with each dome specifically designed to accommodate an oyster and equipped with 25 magnetic sensors. These sensors consist of two components: the HCM1021, a one-axis magnetic sensor from Honeywell, and an offset compensation circuit. They are strategically distributed at varying angles to the base of the half-sphere dome: 6°, 30°, 60°, and one additional sensor placed at 90°. Figure 2e illustrates the arrangement of these sensors. All sensors were affixed to the dome using a cyanoacrylate paste and are encased in a Plexiglas tube for protection against water. All sensors associated with a dome are connected to independent magnetometers with an acquisition card. These magnetometers are then connected to a dedicated computer by an Ethernet cable so that the data can be transferred and processed by software called *Magneto*, which was designed in 2015 and last updated in 2022 by the company Vega Industrie (Avrainville, France). This interface allows for real-time visualization of the sensor values and offers different configurations and parameters for recording the data (Fig. 2d). Special care is taken to avoid any external magnetic fields in the room, as this could distort the acquisitions. This setup enables the performance of eight parallel acquisitions, with sensor values being collected every second. This enhanced precision is crucial for ensuring the reliability of our acquisitions.

To continuously monitor living oysters, a system of water circulation and pump for algae supply was set up. The systems can be adjusted to control the flow of food, water, and temperature. Each dome is supplied with $5\,\upmu \hbox {m}$ filtered seawater continuously. The pearl oysters are fed continuously with a mixture of microalgae consisting of *Isochrysis lutea* and *Chaetoceros gracilis* at a concentration of 30 cells $\upmu \hbox {L}^{-1}$ in each dome. The concentration of microalgae in the experimental domes is checked daily to ensure that the oysters are consistently fed the same amount and that the food doesn’t affect the rotation. The seawater of the domes was renewed at a rate of 7 L.$\hbox {h}^{-1}$. Temperature fluactuated with ambiant temperature (mean 28.2 ± 0.5 °C) Furthermore, the oysters are not affixed to the device but rather allowed to move freely, as depicted in Supplementary Fig. 2.

### Data calibration and performance evaluation

**Figure 3:**
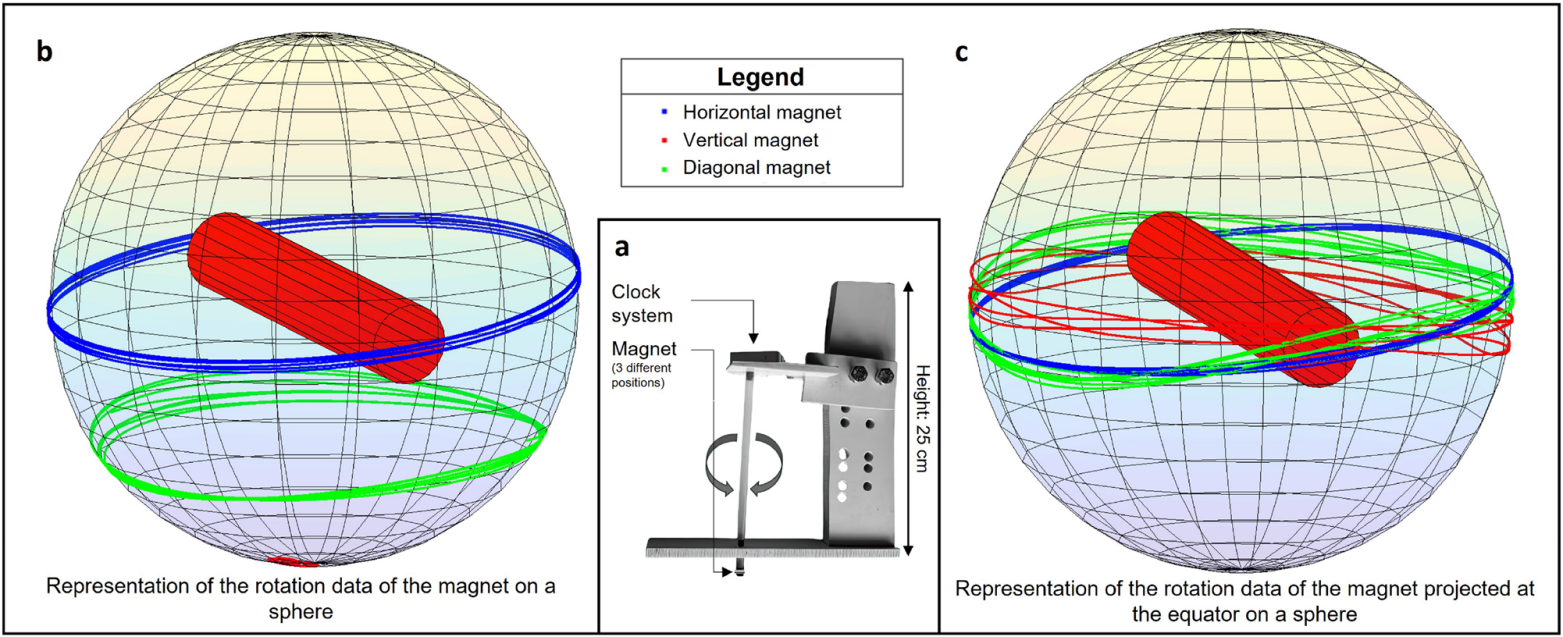
Description of the calibration of our system using a clock system. (**a**) Diagram of the device used: a clock mechanism is fixed at the top of a rod, allowing it to rotate at a fixed speed of one revolution per hour. At the bottom of the rod, a magnet is fixed in a variable position (parallel, perpendicular, or diagonal to the rotation axis). During the acquisition, the magnet is centered at different positions of the dome, in the middle of each of the 3 rows of sensors. (**b**) Representation of the magnetic field data of the magnet projected on a sphere, assimilated to a pearl. (**c**) Representation of the magnetic field data of the magnet projected on the equator, to simulate the real movement of the pearl. The final accuracies are calculated from the projected data at the equator, averaged over the 3 different positions of the magnet at the end of the rod.

To calibrate and ensure the accuracy of our magnetic sensors, we built a calibration device using a clock mechanism and a magnet (Fig. 3). The purpose of the device was to enable us to determine suitable noise filters, optimal oyster positions, and sensor performance. The magnet, which is placed at the end of the rod, is positioned in three different locations within our dome - at the center of each of the sensor lines. The magnet was oriented at three different angles with respect to the axis of rotation - parallel, diagonal, or perpendicular - to evaluate the accuracy of our measurements. By comparing the data from our magnetic field acquisition to the clock’s rotation speed, we calculated the accuracy of our measurements for each sensor line, averaging over the three different orientations of the magnet. After applying a Gaussian-weighted moving average filter with a window length of 60, we achieved accuracies of 97.75%, 98.71%, and 58.5% in the first, second, and third sensor lines, respectively. Thus, an appropriate base was created to place our oysters in the middle of the second row of sensors for optimal measurements. The clock device was critical for calibration as the rotational speed of pearls inside oysters is uncertain, making it challenging to evaluate the measurement accuracy based solely on pearl data.

### Grafts

All donor and recipient oysters were adult individuals with an average shell height of 110mm. Three grafting experiments were conducted with wild pearl oysters, *P. margaritifera* (Linnaeus 1758). The first experiment (n = 47 oysters) was conducted at Ifremer facilities in Vairao, Tahiti. The second experiment (n = 40 oysters) used animals collected and cultured at the Pahai Poe pearl farm on Apataki Atoll, French Polynesia. The third experiment (n = 50 oysters) was conducted at the Tahiti Iti Pearl Farm in Vairao, Tahiti, using animals collected and cultured in the Takapoto atoll. After the grafting process, the grafted pearl oysters were monitored for 1 month to assess nucleus retention. For the last two experiments, after the pearl sac were closed, these pearl oysters were transferred to Ifremer’s facilities either by air or sea. Additionally, a few pearl oysters were specifically chosen in the first experiment for immediate acquisition after grafting, aiming to observe their initial rotational movements during the closure of the pearl sac.

We evaluated post-grafting survival results, related to the quality of the magnetized nuclei, shown in Table 1. The importance of using high-quality magnetized nuclei has been established, as better outcomes were observed with well-crafted or medium-crafted nuclei compared to poor-quality ones, and these outcomes were comparable to those obtained with standard nuclei. To validate these findings, we conducted statistical analyses. The ANOVA revealed that the quality of the nucleus has a significant influence on oyster survival (p = 0.0148). To further examine specific group differences, we utilized Tukey’s HSD Post Hoc Test for pairwise comparisons. The detailed results of the post hoc test can be found in Supplementary Table 1.

Additionally, 25 oysters were lost from multiple causes (death, falling off the string, problems in air transport) during cultivation. Therefore, a total of 52 oysters have been in our magnetometer device over a 1-year timespan. The information and final photos of the corresponding pearls are presented in Supplementary Fig. 1 and Table 2.

**Table 1:** Graft survival after 1 month.

	Number	% Alive	Remaining
(a) Quality of the nucleus grafted			
Excellent	15	60	9
Medium	22	63	14
Poor	10	20	2
(b) Quality of the nucleus grafted			
Medium	20	70	14
Poor	20	0	0
(c) Quality of the nucleus grafted			
Medium	50	76	38

The quality of the nuclei was determined by their irregularity compared to a standard nucleus. All nuclei were reviewed by 3 experts. The grafts were carried out at the following pearl farms: (a) Tahiti Iti Pearl Farm (Teahupoo, Tahiti) (b) Harry’s Pearl Farm (Apataki) (c) IFREMER (Vairao, Tahiti) by Josh, Kamoka Pearl.

### Data processing

To process the colected data, software has been implemented in Matlab, available at https://doi.org/10.5281/zenodo.7872014. The software takes raw data from the sensors as input and filters the noise using a Gaussian-weighted moving average filter. It then calculates and displays the orientation of the magnet over time. The orientation of the magnet at a given time is represented by a 3-dimensional coordinate (XYZ) in a space centered at the center of the pearl. To obtain each of the 3 coordinates, the values of each sensor are multiplied by the relative position of the sensors in the given space, and then summed (see Supplementary Table 3). To transform our magnet rotation data into real rotation data of the pearl, a set of projections is needed. For a detailed description of the entire process, please refer to Supplementary Note 1.

In addition to saving all the orientation data of the magnet and the pearl, images are captured to record the movement from the 3D visualization. Starting from the point of view that maximizes the visible rotation data, through a barycenter calculation, 6 images rotated by 60° are acquired for each acquisition (one pearl, 1 week). These images will then be used to classify the shape of the pearl.

After classification tests, we found out that the first image taken on the 3D representation, which contains the most data, was sufficient, as the addition of the other images introduced noise. Thus, each sample in our dataset, which represents 1 week’s data for one pearl, consists of a single RGB color image of size 224 × 224. Weekly acquisitions were firstly made for practical reasons—the device used must be cleaned every week to maintain favorable conditions for the pearl oysters’ development, and the oysters must be removed from the device for cleaning. As such, it was not possible to acquire continuous rotation data for more than 1 week. More details and examples are provided in the Results section.

### Deep learning classification model

Our classification model receives a 224 × 224 × 3 image as input and produces a probability distribution across our three classes as its output. It was designed to make predictions in two different ways: either sample by sample, corresponding to the rotation of a pearl over a week, or for the entire acquisition period of the pearl’s rotation data. For the latter prediction method, we exported the set of weekly predictions for each pearl and retained the class with the highest frequency. The pearls we analyzed were classified into three distinct categories, which were manually labeled by three experts in the field:

- *Round* includes semi-round and round pearls (27.6%)
- *Atypical* includes baroque, drop, button, and circled pearls (21.3%)
- *Other* includes pearls with no mineral deposits, or very irregular deposits (51.1%)

To determine the amount of rotation data required to predict the final shape of a pearl with precision, we created several datasets in addition to the original one. One dataset comprised data from the last week before the oyster’s sacrifice (with one sample per pearl), while another contained data from the last month before the oyster’s sacrifice (with up to four samples per pearl). Additionally, we constructed a dataset from last week’s data, with data separated by the day, to assess the predictive potential of rotation data over a short 24-h period. Each dataset distribution is described in Supplementary Table 4.

To train the model, we partitioned the datasets into train, validation, and test sets using the repeated holdout technique (n = 100). We carefully separated the data linked to individual pearls to ensure exclusivity to one set, thereby reducing overfitting risks. The class balance across splits was maintained, and we allocated 70% of the data to the train set, 15% to the validation set, and another 15% to the test set. We also carefully considered the disctinction between late and early harvesting of our pearls and made sure to maintain a balanced representation of different harvest dates in all three data splits. Once the conditions were established, individuals were randomly assigned to the three splits, ensuring that each individual was appropriately represented in the test set.

**Figure 4:**
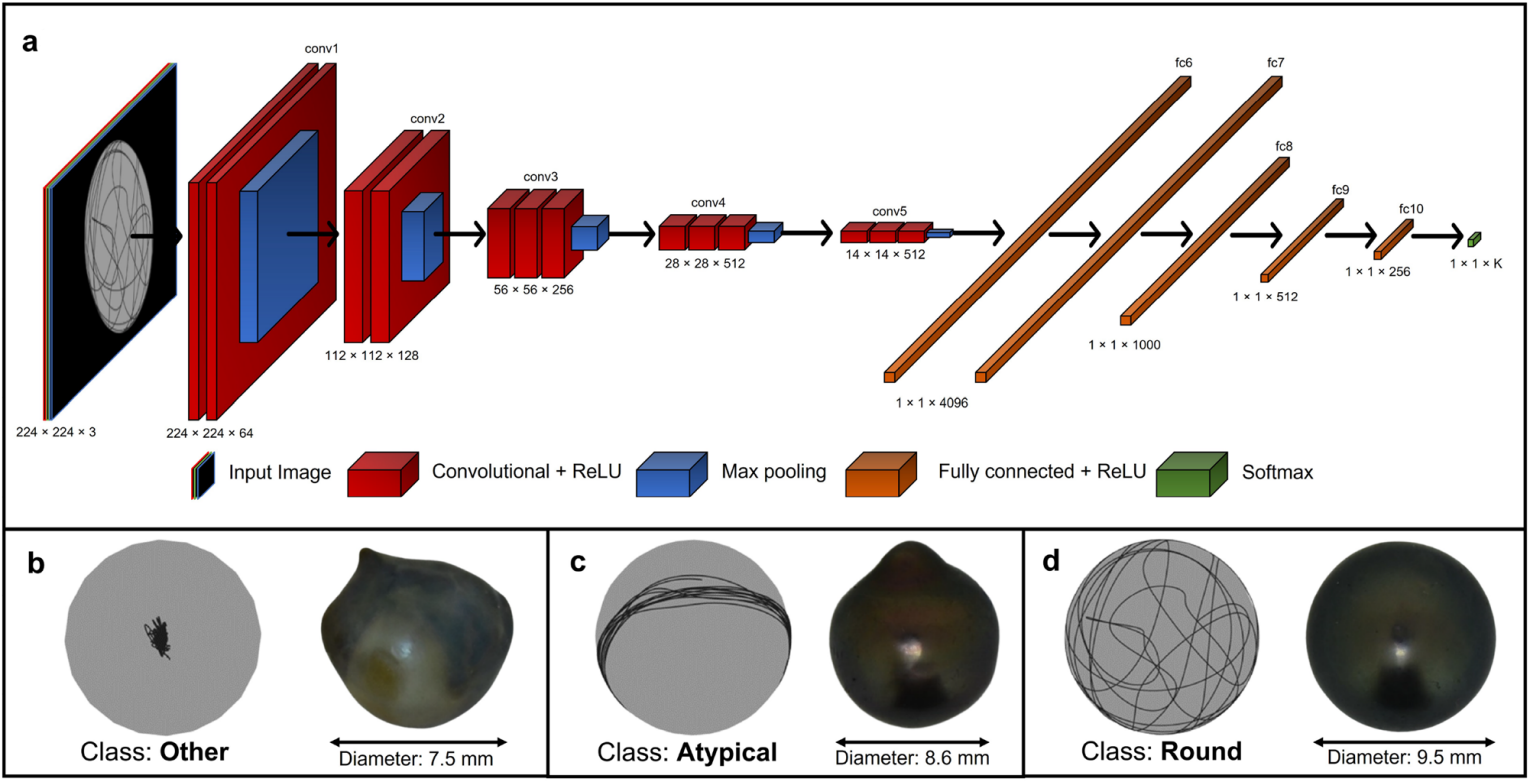
Model architecture and class-associated image and pearl examples. (**a**) Overview of VGG-16 architecture and its component layers (**b**) Example of one image and its corresponding pearl associated with the “Other” class, which includes pearls with no mineral deposits or those with very irregular deposits. (**c**) Example of one image and its corresponding pearl associated with the “Atypical” class, which includes baroque, drop, button, and circled pearls. (**d**) Example of one image and its corresponding pearl associated with the “Round” class, which includes semi-round and round pearls.

To predict the pearl shape using rotation data and account for the limited dataset size, we employed transfer learning with pre-trained neural networks. After evaluating multiple options, presented in the “Material and methods” section, we determined that the VGG-16 convolutional neural network architecture^16^ was the best-suited model for our task, owing to its balance between accuracy and execution time. Previous studies^19,20^ have consistently demonstrated the effectiveness of the VGG-16 model across a diverse range of tasks when compared to alternative architectures. The VGG-16 model is pre-loaded with weights from the ImageNet^15^ dataset and used to extract features from the input images. To use this model, we normalized and resized our datasets images to 224 × 224 × 3. The complete architecture of our VGG-16 model, along with examples from our images, is illustrated in Fig. 4. The architecture includes 18 weight layers and 5 max-pooling layers, each with various functions. A detailed explanation of these functions can be found in Supplementary Note 2.

**Figure 5:**
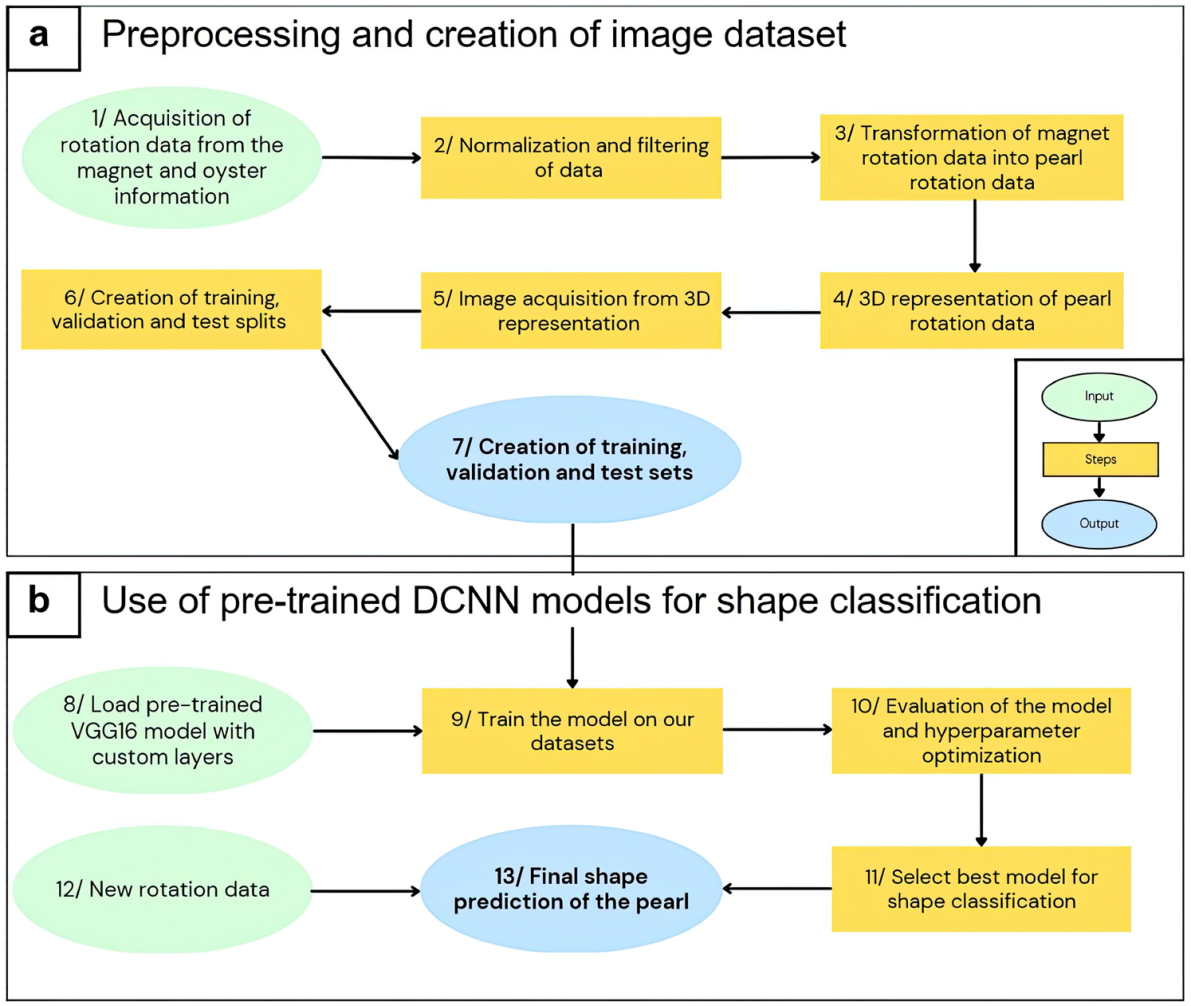
Full description of the entire data handling process, from acquisition to classification. (**a**) Pre-processing and creation of the image dataset, using Matlab^18^. (**b**) Use of pre-trained Deep Convolutional Neural Networks (DCNN) for pearl shape classification using Python and Keras^21^.

After the output of the last max-pooling layer, the output is flattened to add two specific features to each sample with specific weights: the number of days of pearl cultivation until the oyster was sacrificed and the number of days between grafting and rotation acquisition, as rotation is not uniformly distributed during pearl formation. Thanks to an ablation study, it was found that adding these two features resulted in an accuracy gain of approximately 10% for the first feature and 5% for the second. Additional custom layers, including Dense and dropout layers, are then incorporated to prevent overfitting. Finally, the output layer with SoftMax activation is added to classify the pearls’ shapes into three categories. The entire process, from data acquisition to final shape prediction with new data, is summarized in Fig. 5. Each step is elaborated further in Supplementary Note 3.

We evaluated our model’s performance on various datasets using the repeated holdout method with 100 splits. The model accuracy and the weighted-average F1-score were determined for all splits, with associated standard deviations. A grid search was conducted on the training and validation split to optimize the model’s hyperparameters on each dataset and find the best model, and the final hyperparameters are reported Supplementary Table 5. Our main results are summarized and discussed in the Results section.

## Results and discussion

Our goal was to investigate the hypothesis that there exists a correlation between the rotational behavior of a pearl and its final shape. Specifically, we sought to predict the final class of a pearl based on its rotation.

### Machine learning: first classification approach

Initially, we conducted exploratory analysis using conventional machine learning techniques to classify our pearls based on the acquired data. To process the dataset, we calculated features for each sample that captured the pearl’s velocity and acceleration over time. These features were then subjected to binning in order to reduce their overall number. After experimenting with various binning numbers, we determined that selecting 100 features per day was the optimal choice. We utilized the BioDiscML tool^22^ to optimize and evaluate multiple models from our dataset, allowing for effective model comparison. Additionally, we explored the application of LSTM algorithms, which are specifically designed for time-series data analysis, as a means of classification. The outcomes of both approaches are summarized in Supplementary Table 6, highlighting accuracy levels ranging from 20.3 to 51.6%.

All of the above calculations were exclusively performed using the complete dataset and data under matrix form. However, the classification results were significantly unsatisfactory, with the highest achieved accuracy being only 51.6% using a random forest classification approach. As a result of these disappointing outcomes from the previous methods that relied on direct features, our attention shifted towards deep learning methods for image classification. Visual observations of the movement representation served as inspiration for this approach, as they suggested a potential correlation between rotation and form.

### Deep learning: VGG-16 architecture and image classification

We subsequently directed our focus to the VGG-16 architecture and converted our data into the image format, as explained in the “Material and methods” section. Our final dataset consisted of 218 images, with each image depicting a week’s worth of pearl rotation. We removed outliers from the dataset, and a total of 47 distinct pearls were included in the images, with each pearl having a varying number of samples.

**Figure 6:**
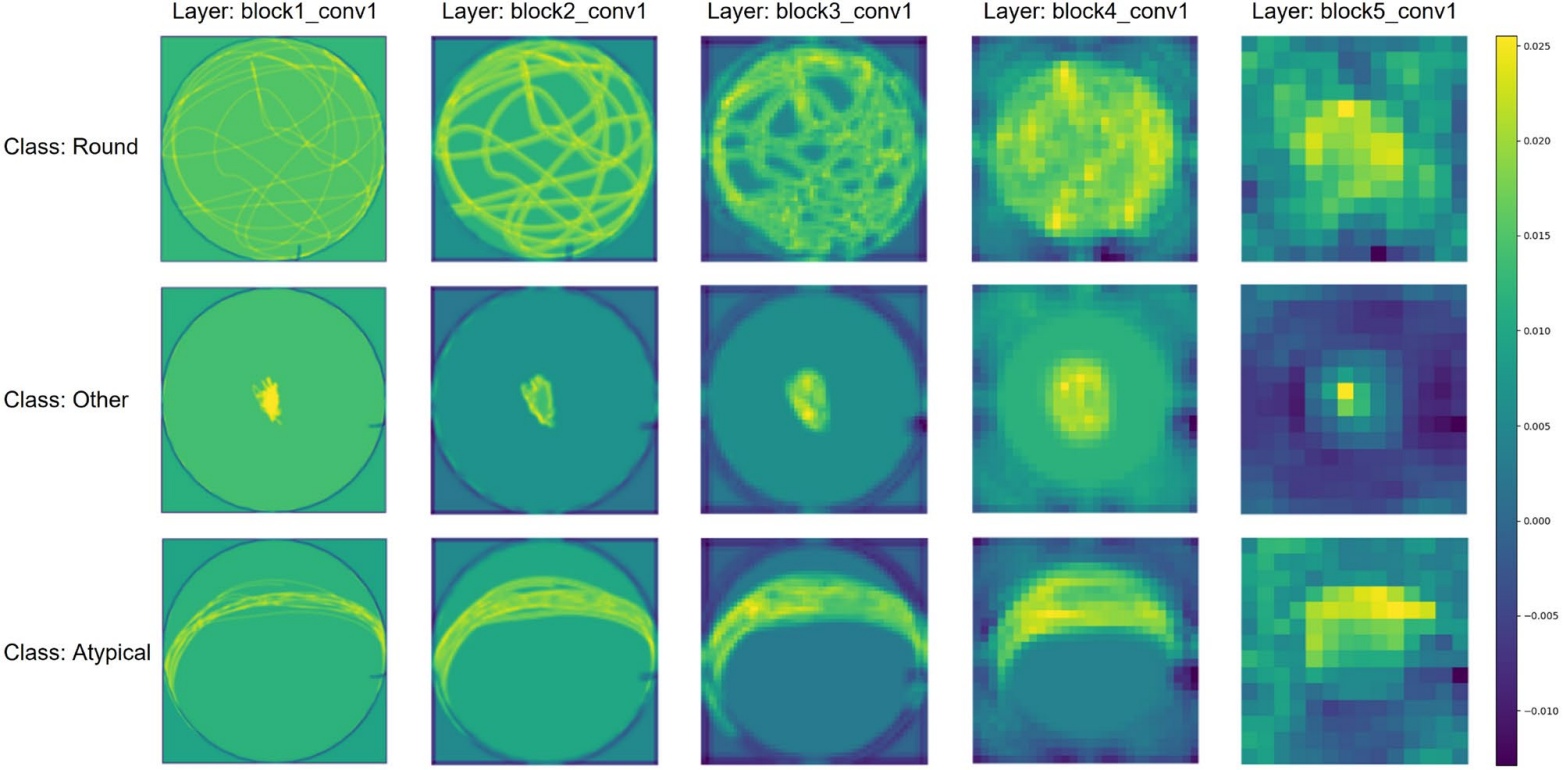
Examples of averaged feature maps for a subset of VGG-16 layers for our three different classes. Feature maps represent the activation values of each filter in a convolutional layer. High activation values (brighter regions in the visualization) indicate that the filter has detected a specific feature in the corresponding region of the input image. Low activation values (darker regions) mean that the filter does not recognize its corresponding feature in that region.

To gain insight into the image processing approach of the VGG-16 architecture, we present in Fig. 6 the results of feature extraction from our images, using the same examples as shown in Fig. 4. The figure displays examples of averaged feature maps for a subset of VGG-16 layers for our three different classes. A comprehensive description of each block is provided in Supplementary Note 4.

From the three displayed images, a clear pattern emerges where a random rotation indicates “Round” pearls, an axial rotation signifies “Atypical” pearls, and no rotation is associated “Other” pearls. These observations confirm the preliminary observations obtained by Gueguen et al.^13^. However, the rotation patterns are more varied than these three categories suggest, and they are often difficult for an observer to classify. This highlights the pertinence of using a classification model.

**Figure 7:**
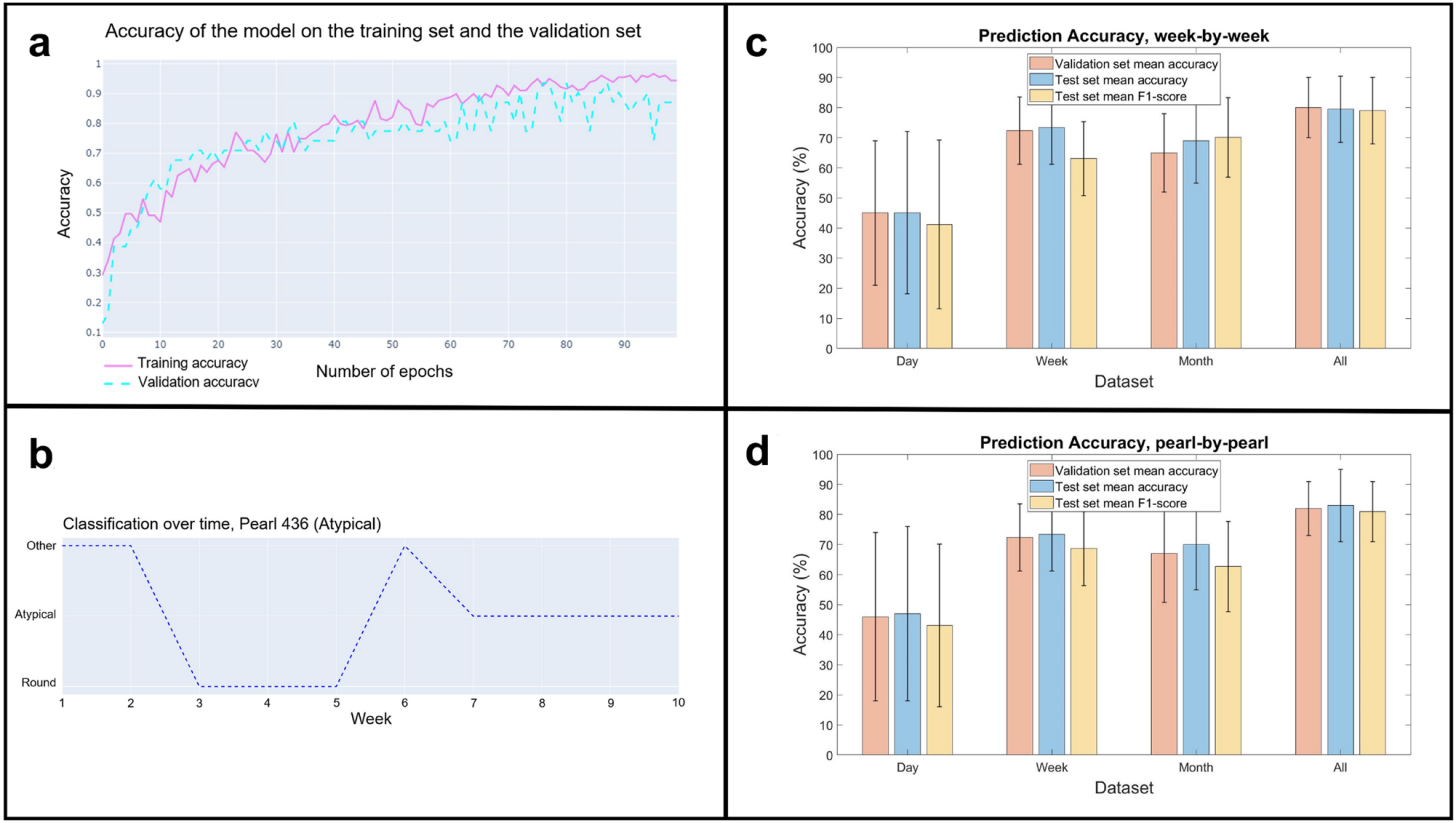
Model evaluation and classification results. (**a**) Example of the evolution of the accuracy on the train (in blue, dotted line) and validation set (in pink, full line) as a function of the number of epochs, used to monitor overfitting. (**b**) Example of classification over time for a pearl. The irregularity of the classification can be explained by the irregularity of the rotation that we observed. (**c**) Different accuracy metrics, on week-by-week classifications, depending on the dataset (day, week, month, all). (**d**) Different accuracy metrics, on pearl-by-pearl classifications, depending on the dataset (day, week, month, all).

The evaluation of the model, as well as all the calculated metrics, are presented in Fig. 7. For the daily, weekly, monthly, and full datasets, we obtained accuracies of 47.1%, 73.4%, 70.1%, and 81.9%, respectively, over the test set and for the final pearl predictions. These results validate that there is a correlation between the pearl’s rotation and its final shape, which can be observed even by analyzing the pearl’s rotation data only from the last week before the oyster’s sacrifice. However, analyzing the rotation daily seems to be insufficient to make a prediction. The calculation of the macro-average F1-score was performed to verify that the prediction is correctly performed regardless of the predicted class and the potential imbalance according to the dataset. Values of 43.1%, 68.7%, 62.7%, and 81% were obtained, for the daily, weekly, monthly, and full datasets, respectively. These high values, except for the 1-day dataset, confirm the high quality of our classification, regardless of the predicted class.The F1-scores for each class have been computed and are presented in Table 2. Despite the imbalance in the monthly and daily datasets, the individual F1 scores show consistency in the weekly and full datasets. These results suggest that our model effectively and accurately classifies all three classes when trained on the weekly or full dataset.

The model achieved the best accuracy when trained on the entire dataset collected over a 1-year period. Our findings confirm that obtaining rotation data throughout the entire pearl formation period improves pearl classification, despite the irregularity of the rotation, compared to using only the last rotation patterns from the final week. In addition, the results indicate that using only the last month of rotation leads to lower performance compared to using either the entire dataset or only the last week.

Although obtaining rotation data from the entire pearl formation period yields the best prediction results, it is not practical to repeat such a lengthy data acquisition for future experiments. The model trained on the weekly dataset achieved an accuracy of 73.4%, allowing shape predictions with just one week of rotation acquisition. We are providing the model trained on our full dataset as a reference for future predictions, regardless of the duration of future acquisitions, and to achieve the best possible prediction. Our model was trained on the data of 47 distinct oysters, from 3 different grafts performed in 3 distinct locations, which reduces the risk of overfitting on a specific graft and should allow reliable predictions for different oysters and grafts.

**Table 2:** F1-score for each class and associated macro-average F1-score, for each dataset.

Dataset	F1−other	F1—atypical	F1—round	Macro-average F1
Day	0.640	0.272	0.382	0.431
Week	0.734	0.672	0.654	0.687
Month	0.787	0.520	0.575	0.627
All	0.826	0.752	0.852	0.81

With an accuracy of 79.5% for weekly classification and 81.9% for bead classification with rotation data acquired over several weeks, our final model establishes a strong correlation between pearl rotation during its formation and its final shape. The provided model and software offer a reliable, turnkey solution for predicting pearl shape from newly acquired rotation data, which can handle input of any size.

### Global observations and measurements of rotation speed

Through our study, we were able to determine the average rotation speed of oysters during pearl formation to be 0.69 ± 0.13° min^−1^ (equivalent to 8.66 ± 1.67 h per revolution), based solely on the rotating pearls during their formation. We also confirmed that rotation should be observed during aragonite deposits on pearls, with 100% of pearls with confirmed deposits having undergone rotation during their formation. However, we did not find a significant difference in rotation speed between pearls of different shapes. Additionally, we observed a sudden acceleration of rotation for two individuals, up to a speed of 4.8° min^−1^, which led to rejection. This observation could provide valuable insight into the mechanism of pearl rejection during formation. To take it a step further, our observations contradict the hypotheses proposed by Cartwright et al.^9^. While we did not observe any deposition without rotation, we did observe rotations occurring without deposition, particularly in the initial stages of the pearl’s growth. This contradicts the initial hypothesis that the deposit of aragonite causes pearl rotation. The initial rotation patterns were identified 21 days after grafting, whereas the first deposits were only visible from the third month onwards. Overall, our findings have important implications for understanding the factors that contribute to successful pearl formation.

### Limitations

Our model currently has some limitations, including the requirement of magnetized nuclei to study rotation. The introduction of a magnet has a weak influence on the graft and final pearl, confirmed by the proportion of round pearls we get, similar to a standard graft^23^, of approximately 30%. Nevertheless, the manual production of these nuclei prevents large-scale studies. Additionally, our rotation measurements do not account for the movement of the oyster during the experiments. Distinguishing rotation along the magnet’s axis from immobility is also challenging. Although our database was acquired over a year, it is small and limited to reliable data from 47 individuals. While a larger study could yield more reliable results, predicting the shape of more than three different classes of pearls would be costly and time-consuming. Moreover, external factors, independent of the rotation, can influence the shape of a pearl. Therefore, we cannot expect significantly higher accuracy than what we currently achieve. Replicating our study is challenging due to the unique nature of each individual and the influence of the timing of data collection relative to the initial grafting date. The methodology presented in the “Material and methods” section provides a framework for reproducing our experimental process, except for the grafting protocol. This aspect is left to the discretion of individual grafters, who maintain confidentiality regarding their specific techniques.

### Future perspectives

The aim of this model and associated acquisition device is to enable non-invasive monitoring of pearl formation through rotation data. Numerous possibilities arise from studying the relationships between rotation and various attributes of the pearl or the oysters that produced it. Additionally, studying the parameters that influence rotation, such as temperature and food, and attempting to link it to the oyster’s muscle activity and respiratory cycle would allow us to identify ideal conditions for controlling the final shape of the pearl after grafting. Studying different patterns and speeds of rotation can help us understand the impact of parameter changes on rotation patterns and speed without sacrificing the oyster. Our observations suggest that a sudden increase in rotation speed could cause rejection, highlighting the need for further research into the rejection mechanism. Understanding this mechanism could potentially help prevent rejection from occurring, leading to significant improvements in the control and quality of pearl production. Therefore, it is important to gain an extensive understanding of the impact of rotation on pearl attributes to advance research in this area.

### Conclusion

In conclusion, this study has confirmed the correlation between rotation and the final shape of the pearl, as well as the capital importance of the rotation in the creation and the deposit of aragonite on the nucleus. This study also introduced a device that enables non-invasive monitoring for scientific research on pearls. This device allows for accessible and small-scale studies on parameters that can affect pearl formation and its final attributes. Compared to conventional methods, which require waiting for the entire pearl production process (12−18 months) to study parameter influences, the non-invasive monitoring offered by our device over any short period of time offers a more accessible approach.

### Supplementary Information

Supplementary Information.

## Data Availability

The data that supports the findings of this study and used to train the given model are available from the corresponding author upon reasonable request.

## Code availability

The codes for processing rotation data and predicting pearl shape are available online: https://doi.org/10.5281/zenodo.7872014. All other codes used in our study, especially for training our model, are available from the corresponding author upon reasonable request.

## References

[1] Direction des Ressources Marines de la Polynésie Française. *Bulletin Statistique de la Direction des Ressources Marines de la Polynésie Française*. DRM (2021).

[2] Yukihira H, Lucas JS, Klumpp DW (2006). The pearl oysters, pinctada maxima and p. margaritifera, respond in different ways to culture in dissimilar environments. Aquaculture.

[3] Southgate, P. & Lucas, J. *The Pearl Oyster* 272–302 (Elsevier, 2008).

[4] Jameson HL (1902). On the origin of pearls. Proc. Zool. Soc. Land..

[5] Wada K (1999). Formation and quality of pearls. J. Genmol. Soc. Jpn..

[6] Gueguen, Y. *et al*. Characterization of molecular processes involved in the pearl formation in *Pinctada margaritifera* for a sustainable development of pearl farming industry in French Polynesia. *Recent Advances in Pearl Research* 183–195 (2013).

[7] Caseiro J (1995). Evolution de l’épaisseur des dépots de matériaux organiques et aragonitiques durant la croissance des perles de pinctada margartitifera. CR Acad. Sci. Paris Sér.

[8] Linard C (2011). Calcein staining of calcified structures in pearl oyster *Pinctada margaritifera* and the effect of food resource level on shell growth. Aquaculture.

[9] Cartwright JH (2013). Pearls are self-organized natural ratchets. Langmuir.

[10] Ky CL (2018). Phenome of pearl quality traits in the mollusc transplant model *Pinctada margaritifera*. Sci. Rep..

[11] Le Luyer J (2019). Whole transcriptome sequencing and biomineralization gene architecture associated with cultured pearl quality traits in the pearl oyster, *Pinctada margaritifera*. BMC Genom..

[12] Ky CL (2018). The mendelian inheritance of rare flesh and shell colour variants in the black-lipped pearl oyster (*Pinctada margaritifera*). Anim. Gen..

[13] Gueguen Y (2015). Yes, it turns: experimental evidence of pearl rotation during its formation. R. Soc. Open Sci..

[14] Le Moullac G (2018). Influence of temperature and pearl rotation on biomineralization in the pearl oyster, pinctada maragaritifera. J. Exp. Biol..

[15] Russakovsky O (2015). Imagenet large scale visual recognition challenge. Int. J. Comput. Vis..

[16] Simonyan, K., & Zisserman, A.. Very deep convolutional networks for large-scale image recognition. *Computer Vision and Pattern Recognition* (2015).

[17] Cochennec N (2010). A histological examination of grafting success in pearl oyster *Pinctada margaritifera* in French Polynesia. Aquat. Living Resour..

[18] The MathWorks Inc. Matlab version: 9.13.0 (r2022b) (2022).

[19] Wei, Y. *et al*. Visualizing and comparing alexnet and vgg using deconvolutional layers. *ICML 2016 Workshop on Visualization for Deep Learning* (2016).

[20] Kaur, T., & Gandhi, T. K. Imagenet large scale visual recognition challenge. * 2019 International Conference on Information Technology* 94–98 (2019).

[21] Abadi, M. *et al*. TensorFlow: Large-scale machine learning on heterogeneous systems. Software available from tensorflow.org. (2015).

[22] Leclercq M (2019). Large-scale automatic feature selection for biomarker discovery in high-dimensional omics data. Front. Genet..

[23] Ky CL (2015). Influence of grafter skill and season on cultured pearl shape, circles and rejects in *Pinctada margaritifera* aquaculture in mangareva lagoon. Aquaculture.

