# Supplementary material for: Pearl shape classification using deep convolutional neural networks from Tahitian pearl rotation in Pinctada margaritifera
Source: Sci Rep. 2023 Aug 12;13:13122. doi: 10.1038/s41598-023-40325-z (PMC10423288; doi:10.1038/s41598-023-40325-z)
Supplement: Supplementary file 1 — Supplementary Information. [file 41598_2023_40325_MOESM1_ESM.pdf]

Supplementary Materials

**Group 1**	**Group 2**	**MeanDiff**	**P-adj**	**Lower**	**Upper**	**Correlation**
Excellent	Medium	9.6667	0.6979	-37.3357	56.6691	**False**
Excellent	Poor	-50	0.0496	-99.8536	-0.1464	**True**
Medium	Poor	-59.6667	0.0138	-96.8253	-22.508	**True**

Table 1: Multiple Comparison of Mean -Tukey HSD, FWER = 0.05

Oyster	Dome	Graft Date	Acq. Start Date	Acq. End Date	Shape	Diameter (cm)
639	1	02/12/2021	02/12/2021	01/03/2022	Atypical	7.85
640	2	02/12/2021	02/12/2021	26/01/2022	Other	7.33
643	5	02/12/2021	02/12/2021	26/01/2022	Other	7.52
644	6	02/12/2021	02/12/2021	16/12/2021	Other	None
645	9	02/12/2021	02/12/2021	26/01/2022	Other	7.24
646	10	02/12/2021	02/12/2021	26/01/2022	Other	7.34
642	4	02/12/2021	02/12/2021	10/08/2022	Round	8.12
641	5	02/12/2021	02/12/2021	10/08/2022	Atypical	7.79
27	5	02/12/2021	26/01/2022	01/02/2022	Other	6.92
28	9	02/12/2021	26/01/2022	19/04/2022	Other	None
29	10	02/12/2021	26/01/2022	19/04/2022	Other	None
26	2	02/12/2021	26/01/2022	10/08/2022	Atypical	8.08
25	1	02/12/2021	01/02/2022	07/03/2022	Other	7.48
644_2	5	02/12/2021	01/03/2022	19/04/2022	Atypical	None
640_2	1	02/12/2021	07/03/2022	18/05/2022	Atypical	8.26
717	10	02/12/2021	19/04/2022	18/05/2022	Other	8.18
400	9	02/12/2021	19/04/2022	18/05/2022	Other	7.62
25_2	1	02/12/2021	18/05/2022	31/05/2022	Other	None
27_2	9	02/12/2021	18/05/2022	31/05/2022	Other	3.91
188	10	02/12/2021	18/05/2022	10/08/2022	Atypical	7.98
903	1	02/12/2021	31/05/2022	09/06/2022	Atypical	7.95
904	9	02/12/2021	31/05/2022	10/08/2022	Atypical	7.85
905	1	02/12/2021	09/06/2022	14/06/2022	Other	7.72
906	1	02/12/2021	14/06/2022	10/08/2022	Atypical	7.88
907	1	25/11/2021	10/08/2022	18/08/2022	Round	8.41
908	2	25/11/2021	10/08/2022	18/08/2022	Other	8.71
909	4	25/11/2021	10/08/2022	18/08/2022	Other	9
910	10	01/03/2022	10/08/2022	18/08/2022	Atypical	8.36
911	5	01/03/2022	10/08/2022	18/08/2022	Other	6.59
912	10	01/03/2022	11/08/2022	18/08/2022	Other	7.47
913	1	01/03/2022	18/08/2022	11/10/2022	Other	7.27
914	2	01/03/2022	18/08/2022	26/09/2022	Other	7.75
915	4	01/03/2022	18/08/2022	11/10/2022	Other	7.12
916	5	01/03/2022	18/08/2022	11/10/2022	Other	8.31
917	9	01/03/2022	18/08/2022	11/10/2022	Round	9.13
918	10	01/03/2022	18/08/2022	26/09/2022	Other	7.76
919	2	01/03/2022	26/09/2022	03/11/2022	Other	7.44
920	10	01/03/2022	26/09/2022	17/10/2022	Other	8.17
921	1	01/03/2022	11/10/2022	03/11/2022	Other	7.61
922	4	01/03/2022	11/10/2022	03/11/2022	Round	8.91
923	5	01/03/2022	11/10/2022	21/10/2022	Other	6.95
924	9	01/03/2022	11/10/2022	03/11/2022	Other	6.94
925	10	01/03/2022	17/10/2022	03/11/2022	Round	8.94
926	1	01/03/2022	03/11/2022	18/11/2022	Round	8.94
927	2	01/03/2022	03/11/2022	18/11/2022	Round	8.29
928	4	01/03/2022	03/11/2022	18/11/2022	Round	9.09
929	9	01/03/2022	03/11/2022	18/11/2022	Round	8.69
930	10	01/03/2022	18/11/2022	24/11/2022	Round	8.18
931	1	01/03/2022	18/11/2022	24/11/2022	Round	8.78
932	2	01/03/2022	18/11/2022	24/11/2022	Atypical	7.85
933	4	01/03/2022	18/11/2022	24/11/2022	Atypical	8.4
934	9	01/03/2022	18/11/2022	24/11/2022	Other	None
436	6	02/12/2021	16/12/2021	24/11/2022	Round	8.93

Table 2: Table including all the information associated with each oyster: the date of grafting, the start and end dates of acquisition, as well as the final shape attributed. Note that the end date of acquisition also corresponds to the date of sacrifice for the oyster.

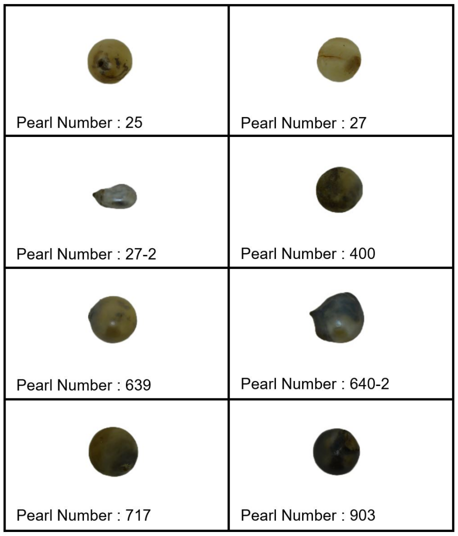
Pearl Number : 25
Pearl Number : 27
Pearl Number : 27-2
Pearl Number : 400
Pearl Number : 639
Pearl Number : 640-2
Pearl Number : 717
Pearl Number : 903

(a)

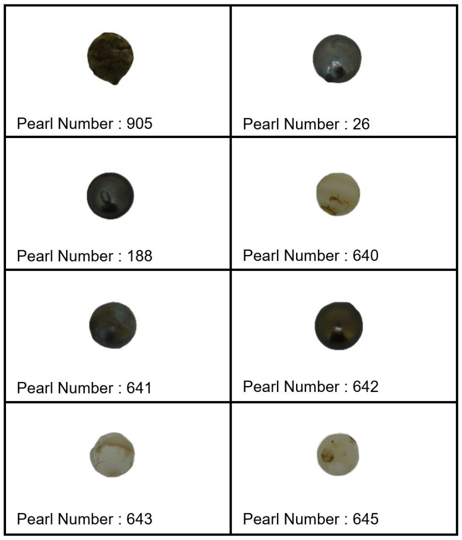
Pearl Number : 905
Pearl Number : 26
Pearl Number : 188
Pearl Number : 640
Pearl Number : 641
Pearl Number : 642
Pearl Number : 643
Pearl Number : 645

(b)

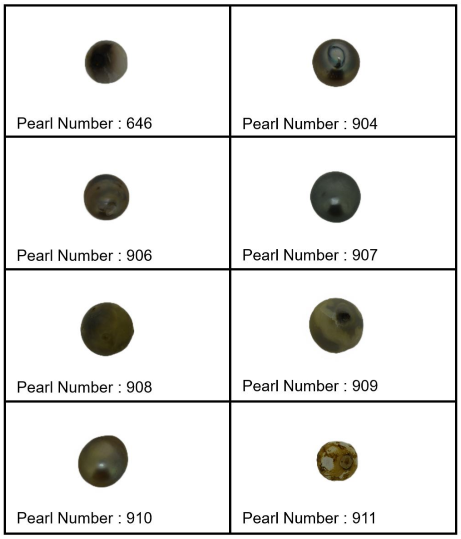
Pearl Number : 646
Pearl Number : 904
Pearl Number : 906
Pearl Number : 907
Pearl Number : 908
Pearl Number : 909
Pearl Number : 910
Pearl Number : 911

(c)

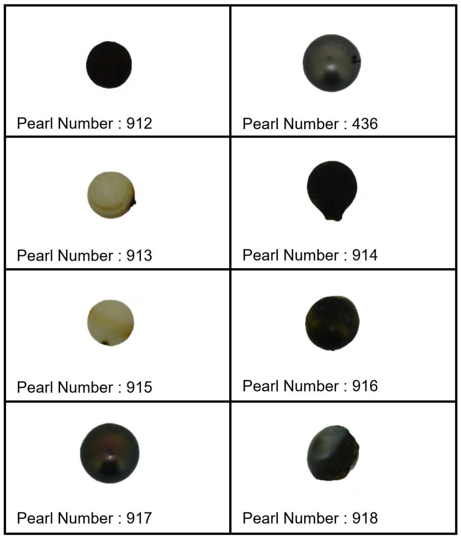
Pearl Number : 912
Pearl Number : 436
Pearl Number : 913
Pearl Number : 914
Pearl Number : 915
Pearl Number : 916
Pearl Number : 917
Pearl Number : 918

(d)

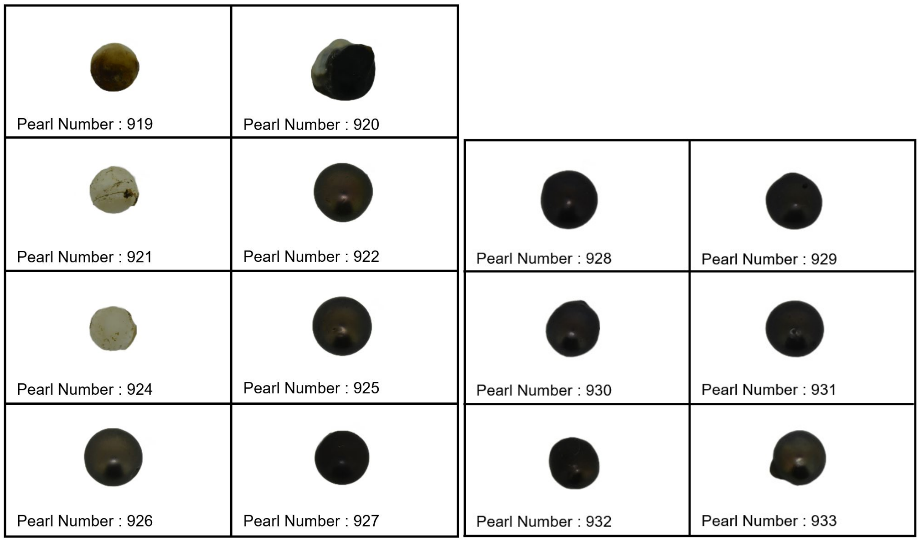

(e) (f)

Figure 1: Photographs of all the pearls that were processed by our device are included, except for the 7 pearls that were lost between the characterization and photography stages.

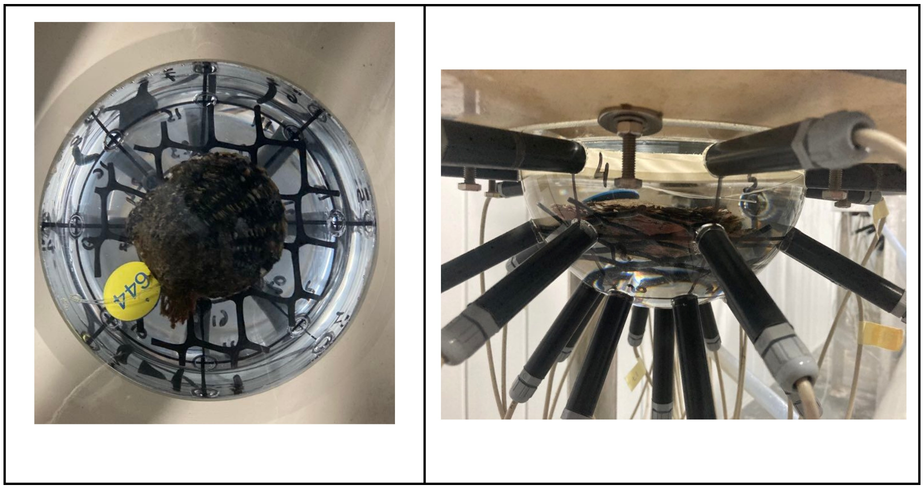

Figure 2: Representation of an oyster's position in a dome, viewed from above and from the side.

Sensors	C1	C2	C3	C4	C5	C6	C7	C8
X	0.9945	0.7032	0	-0.7320	-0.9945	-0.7032	0	0.7032
Y	0	0.7032	0.9945	0.7032	0	-0.7032	-0.9945	-0.7032
Z	0.1045	0.1045	0.1045	0.1045	0.1045	0.1045	0.1045	0.1045

Sensors	C9	C10	C11	C12	C13	C14	C15	C16
X	0.8660	0.6124	0	-0.6124	-0.8660	-0.6124	0	0.6124
Y	0	0.6124	0.8660	0.6124	0	-0.6124	-0.8660	-0.6124
Z	0.5	0.5	0.5	0.5	0.5	0.5	0.5	0.5

Sensors	C17	C18	C19	C20	C21	C22	C23	C24
X	0.5	0.3536	0	-0.3536	-0.5	-0.3536	0	0.3536
Y	0	0.3536	0.5	0.3536	0	-0.3536	-0.5	-0.3536
Z	0.8660	0.8660	0.8660	0.8660	0.8660	0.8660	0.8660	0.8660

Sensors	C25
X	0
Y	0
Z	1

Table 3: Cartesian coordinates of each magnetic sensor.

**Class**	**Daily instances**	**Weekly instances**	**Monthly instances**	**All instances**
Other	168	24	52	127
Atypical	70	10	29	117
Round	91	13	29	114

Table 4: Number of instances for each class in each dataset. ”Daily” dataset corresponds to the weekly dataset, but with data subdivided by the day. This means that each instance in the ”Daily” dataset represents one day’s worth of data, whereas in the other datasets, each instance represents one week’s worth of data. The imbalance is caused by the different acquisition times from one pearl to another.

**Hyperparameter**	**Value**
Epochs	100
Batch Size	16
Learning Rate	1.0e-4
Optimizer	Adam
Pre-trained weights	ImageNet
Layers Frozen	First 16 layers
Loss Function	Categorical Cross-Entropy

Table 5: Hyperparameters for VGG-16 Model

Model	Best Accuracy Obtained
Random Forest	0.515
Naive Bayes	0.468
Simple Logistic Regression	0.422
RBF Network	0.390
OneR	0.203
LSTM	0.441

Table 6: Performance results from various models on our dataset. The best accuracy achieved after optimization is reported, with each accuracy score computed on the test set and represented as the mean of randomized batches. Special care is taken to split oysters into different sets to avoid overfitting. The BioDiscML [22] tool was utilized to calculate all models, with the exception of the LSTM model, which was manually coded and optimized in Python. Bidirectional LSTM model was also tried, giving the same results as LSTM model. This table gives an overview of all the results obtained, but it does not include all the models tested. Details about the parametrization of these models can be found at https://github.com/mickaelleclercq/BioDiscML/blob/master/classifiers.conf

Supplementary Note 1: Transformation of our magnet rotation data into real rotation data of the pearl

Let M_{magnet} be the matrix containing all the magnet orientation data over time, of size $(T_{tot}, 3)$. The following are the steps to create the new matrix M_{pearl}, which corresponds to the orientation data of the pearl, treated as a sphere:

1. For a 3D point $M_{magnet}(t)$, which represents the magnet orientation at a certain time, determine two other points at the same time interval: $M_{magnet}(t - t_{pearl})$ and $M_{magnet}(t + t_{sphere})$. In our case, we chose $t_{sphere} = 1600$ seconds, considering the average rotation speed of our pearls.

2. Calculate the equation of the plane (P1) passing through these three points.

3. Determine the equation of the plane (P2), parallel to the plane (P1) and passing through the origin of the sphere.

4. Determine the plane (P3), perpendicular to both planes (P1) and (P2) and passing through the point $M(t, :)$.

5. Calculate the line (D1) that intersects both planes (P2) and (P3).

6. Calculate the two points $(M^1_{sphere}(t))$ and $(M^2_{sphere}(t))$ where line (D1) intersects the sphere representing the pearl. Then, calculate the distances between $M_{magnet}(t)$ and these two points, and keep the point of intersection that corresponds to the minimal distance, giving the final point $M_{sphere}(t)$.

To obtain the new matrix M_{sphere}, which corresponds to the orientation data of the pearl over time, repeat the six steps outlined above for each point in the matrix M_{magnet}. The rotation of the pearl over time is finally determined, allowing for the computation of its corresponding velocities and accelerations.

Supplementary Note 2: Detailed description of our VGG-16 architecture

1. Input layer: This layer receives the RGB image input, which is resized to 224x224 pixels.

2. Convolutional layers: The VGG-16 architecture comprises 13 convolutional layers, each with a 3x3 kernel. These layers extract features from the input image. The number of filters in the convolutional layers increases with depth, starting from 64 and doubling after each max-pooling layer until it reaches 512.

3. Max-pooling layers: VGG-16 includes 5 max-pooling layers, each using a 2x2 kernel with a stride of 2. These layers help to reduce the spatial dimensions of the feature maps, thereby decreasing computational complexity and capturing translation-invariant features. They are interspersed between the convolutional layers.

4. Fully connected layers: After the convolutional and max-pooling layers, VGG-16 has 3 fully connected (dense) layers. The first two dense layers have 4096 units each, followed by three layers with decreasing unit sizes of 1000, 512, and 256, respectively. The size of the final dense layer depends on the number of output classes K in the classification task, which is 3 in our case. These layers integrate the high-level features extracted by the convolutional layers to make the final classification decision. Each fully connected layer is followed by a 50% dropout layer to prevent overfitting.

5. Activation functions: The VGG-16 architecture employs the Rectified Linear Unit (ReLU) activation function throughout the network, except for the final dense layer, where a SoftMax activation function is utilized to output class probabilities.

6. Output layer: The VGG-16 network's output layer provides the class probabilities, and the class with the highest probability is chosen as the final classification result.

Supplementary Note 3: Detailed description of the entire data handling process, from acquisition to classification.

1. Input: acquisition of rotation data from the magnet, and oyster information (number of days between graft and acquisition). One sample is equal to one week of rotation acquisition for the pearl.

2. Normalization and filtering of data using a Gaussian-weighted moving average filter.

3. Transformation of magnet rotation data into pearl rotation data, using projections to the equator of a sphere, as described in the Material and Methods section.

4. 3D representation of pearl rotation data, by assimilating the pearl to a sphere.

5. Image acquisition from prior 3D representations, creating 224x224 RGB images of data projected onto a sphere centered on the data's barycenter.

6. Creation of training, validation, and test sets using the Repeated Holdout method with a 70%/15%/15% split.

7. Output: Training, validation, and test sets are created from filtered, consistent data with added metadata (time elapsed since grafting for acquisition and sacrifice). After filtering outliers due to various acquisition issues, a total of 218 samples from 47 different pearls were retained.
8. Input: Load a pre-trained VGG16 model with custom layers, using weights pre-trained on the ImageNet [20] dataset.
9. Training of the model on our datasets to fine-tune the weights for our images.
10. Hyperparameter optimization using grid search on batch size, learning rate, epochs, and regularization. Evaluation of different models from accuracy and f1-score.
11. Selection of the best model for shape classification on our pearls
12. Input: New rotation data transformed into images as in the training set to be used on our trained model.
13. Output: Final shape prediction of the pearl from new rotation data.

Supplementary Note 4: Detailed description of each block from the feature extraction process, from Fig. 6.

1. Block1-conv1: This is the first layer of the VGG-16 model that detects low-level features such as edges and textures.
2. Block2-conv1: This is the first layer of the second block, where the model starts recognizing more complex patterns like corners and simple shapes.
3. Block3-conv1: The first layer of the third block captures higher-level features, such as object parts and more complex shapes.
4. Block4-Conv1: In the first layer of the fourth block, the model identifies even more abstract features, like parts of objects or specific textures related to the objects in the image.
5. Block5-conv1: The first layer of the fifth block represents the highest level of abstraction in the VGG-16 model. At this point, the model has captured more complex features and patterns that help it differentiate between various objects and scenes. Visualizing this layer offers insights into the model's ability to recognize high-level visual concepts.
